# Impact of a Multidomain Outdoor Exercise Intervention on Cardiovascular Health and Functional Capacity for Healthy Aging: A Randomized Controlled Trial (ACTIVA-Senior Study)

**DOI:** 10.3390/healthcare13161975

**Published:** 2025-08-12

**Authors:** Antonio Manuel García-Llorente, Dalmo Roberto Lopes Machado, Antonio Jesús Casimiro-Andújar, Pablo Jorge Marcos-Pardo

**Affiliations:** 1 SPORT Research Group (CTS-1024), CIBIS (Centro de Investigación para el Bienestar y la Inclusión Social) Research Center, University of Almeria, 04120 Almeria, Spain; 2 School of Physical Education and Sport of Ribeirão Preto, University of São Paulo, Ribeirão Preto 05508, SP, Brazil; 3 Active Aging, Exercise and Health/HEALTHY-AGE Network, Consejo Superior de Deportes (CSD), Ministry of Culture and Sport of Spain, 28004 Madrid, Spain; 4 Department of Education, Faculty of Education Sciences, University of Almeria, 04120 Almeria, Spain

**Keywords:** multidomain intervention, healthy aging, cardiovascular health, outdoor exercise, blood pressure, functional capacity

## Abstract

**Background**: Population aging calls for effective, scalable interventions to enhance cardiovascular and functional health in older adults. **Objective**: The objective was to evaluate the effects of an 18-week multidomain outdoor exercise program on cardiovascular and functional outcomes. **Methods**: Fifty-two participants (mean age: 66.0 ± 5.1 years; BMI: 27.2 ± 3.7 kg/m^2^; body fat: 35.0 ± 7.0%) were randomized into intervention (n = 26) and control (n = 26) groups. The intervention involved twice-weekly, 60 min outdoor sessions integrating resistance (30–60 s isometric holds), aerobic training (Borg RPE 6–7), balance, and cognitive exercises. **Results**: Forty-six participants completed the study (intervention: n = 25; control: n = 21). The intervention group (mean age 65.6 ± 5.3) demonstrated significant improvements compared to the controls (mean age 66.4 ± 4.9): a systolic blood pressure reduction of 17.4 mmHg (95% CI: −21.9 to −12.9, *p* < 0.001, ηp^2^ = 0.376), diastolic blood pressure reduction of 9.2 mmHg (95% CI: −12.1 to −6.3, *p* < 0.001, ηp^2^ = 0.248), and Six-Minute Walk Test improvement of 64.7 m (95% CI: 45.9 to 83.5, *p* < 0.001, ηp^2^ = 0.463). Body composition showed modest but significant improvements in percent body fat (−1.3%, *p* = 0.007) and visceral fat levels (−0.9 units, *p* = 0.002). However, these changes were not significant between groups. The Number Needed to Treat was 2.2 for clinically significant blood pressure reduction and 1.4 for meaningful functional improvement. **Conclusions**: The ACTIVA-Senior multidomain outdoor intervention led to clinically meaningful improvements in cardiovascular function and functional capacity. The results suggest strong potential for scalable outdoor aging interventions. These findings support the integration of structured outdoor exercise programs into healthy aging strategies and public health initiatives.

## 1. Introduction

Population aging represents one of the most significant demographic transitions of the 21st century, with individuals over 60 years projected to comprise 22% of the global population by 2050 [1]. This demographic shift presents unprecedented challenges for healthcare systems, particularly regarding cardiovascular disease management, which remains the leading cause of morbidity and mortality in older adults [2]. Cardiovascular diseases, including hypertension and arterial stiffness, affect over 70% of adults aged 65 and older, contributing to functional decline, disability, and increased healthcare costs [3]. The prevalence of hypertension increases dramatically with age, affecting nearly 90% of individuals over 80 years [2]. Moreover, age-related cardiovascular changes, including reduced arterial compliance and endothelial dysfunction, compound the risks associated with sedentary lifestyles and multimorbidity [4].

Physical activity is a well-established non-pharmacological intervention for preventing and managing cardiovascular and metabolic disorders [5,6]. Regular exercise improves endothelial function, arterial compliance, and autonomic blood pressure regulation, contributing to reduced cardiovascular risk [7,8]. Exercise also improves body composition by reducing fat mass and preserving lean muscle, which counteracts sarcopenia [9,10]. These benefits enhance muscle strength, balance, and mobility, critical factors for fall prevention and functional independence [11]. Additionally, structured exercise provides cognitive benefits, supporting autonomy and reducing disability risk [12]. Multidomain interventions, which integrate physical exercise with cognitive training and lifestyle modifications, represent an innovative approach to healthy aging [9,10]. Cognitive flexibility—the capacity to shift between tasks, strategies, or mental sets—is a core component of executive function that declines with age, contributing to slower learning, reduced problem-solving ability, and diminished capacity to manage complex daily activities [9]. Preserving this ability is essential for maintaining independence, social participation, and overall quality of life in older adults, and may also enhance their capacity to benefit from physical training.

Large-scale trials, including the Finnish Geriatric Intervention Study to Prevent Cognitive Impairment and Disability (FINGER) and the Korean SUPERBRAIN study, have demonstrated the superior efficacy of multidomain approaches compared to single-domain interventions [13,14]. These studies reveal that comprehensive programs addressing multiple physiological systems simultaneously produce greater benefits than isolated interventions. Additionally, individuals undertaking multidomain sessions experienced a reduced incidence of total cardiovascular events (Hazard ratio = 0.50, 95% CI: 0.28–0.90) and stroke/transient ischemic attack (Hazard ratio = 0.40, 95% CI: 0.20–0.81) compared with those in the control group [14].

Isometric exercise training (IET) has gained particular attention for blood pressure management in older adults [15]. Systematic reviews demonstrate that IET produces superior reductions in both systolic and diastolic blood pressure compared to traditional aerobic or resistance training alone [16]. The mechanisms underlying IET effectiveness include enhanced endothelium-dependent vasodilation, improved baroreflex sensitivity, and reduced peripheral vascular resistance [17]. Critically, blood pressure reductions as modest as 5 mmHg are associated with 10–15% reductions in cardiovascular events and mortality [3].

Despite growing evidence supporting multidomain interventions, most research has focused on facility-based or home-based programs [18]. Outdoor exercise environments offer unique advantages, including enhanced psychological well-being, improved adherence, vitamin D synthesis, and exposure to natural settings that may amplify intervention benefits [19,20]. Furthermore, outdoor programs present scalable, cost-effective solutions suitable for community implementation and public health initiatives [20]. The Six-Minute Walk Test (6MWT) serves as a validated measure of cardiovascular endurance and functional capacity in older adults [21]. The minimal clinically important difference for 6MWT performance in Asian older adult populations is established at 17.8 m, with improvements exceeding this threshold correlating with reduced cardiovascular risk and enhanced quality of life [22].

This study addresses a critical gap in the literature [23] by evaluating the cardiovascular and functional effects of a structured 18-week multidomain outdoor exercise intervention in healthy older adults. While existing programs are often limited to facility- or home-based settings, outdoor approaches offer distinct advantages—including accessibility, cost-effectiveness, and mental health benefits. Our comprehensive program, which combines isometric exercises, aerobic training, balance work, and cognitive tasks, was designed to be scalable and holistic. We hypothesized that this intervention would lead to clinically meaningful improvements in blood pressure regulation and functional capacity compared to control conditions, with potential implications for national aging policy and the promotion of long-term cardiovascular and functional health.

## 2. Materials and Methods

### 2.1. Study Design and Participants

This single-blind randomized controlled trial followed CONSORT 2025 recommendations and was approved by the University of Almería Ethics Committee (ID: 3322/2021). The study was prospectively registered at ClinicalTrials.gov (NCT05481346).

**Figure 1:**
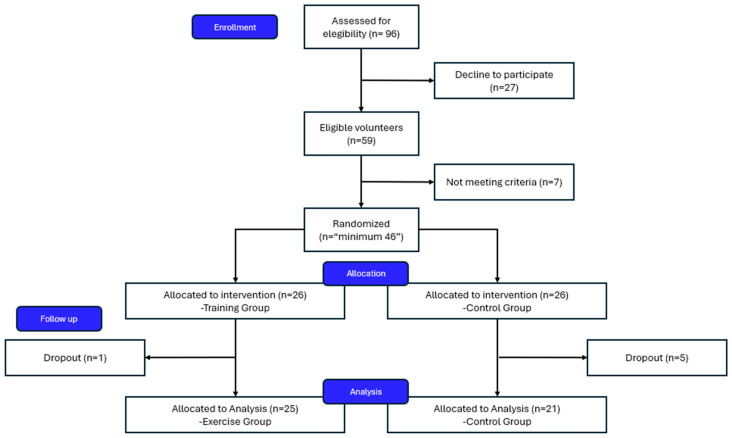
Flowchart of participant selection, allocation, follow-up, and analysis in the ACTIVA-Senior study.

A participant flow diagram aligned with CONSORT guidelines is provided in Figure 1 (and the CONSORT checklist can be found in the Supplementary File). More detailed information should be provided in the ACTIVA-Senior Protocol Study [24].

*The inclusion criteria were* (1) age ≥ 60 years; (2) written medical clearance from a primary care physician or specialist, confirming ability to safely engage in moderate-to-vigorous physical activity; (3) no diagnosed contraindications to physical activity as per the American College of Sports Medicine guidelines [25], including unstable cardiovascular, pulmonary, or metabolic conditions; (4) cognitive capacity to provide informed consent and follow instructions; and (5) availability to attend twice-weekly sessions for 18 weeks. *The exclusion criteria included* (1) uncontrolled hypertension (>180/110 mmHg); (2) history of recent cardiovascular events (<6 months); (3) advanced-stage malignancy; (4) musculoskeletal or neurological conditions (e.g., severe osteoarthritis, recent fracture, advanced Parkinson’s disease) that would preclude safe performance of the prescribed exercises; (5) cognitive impairment (Mini-Mental State Examination < 24); and (6) participation in structured exercise programs > 150 min weekly.

### 2.2. Sample Size Calculation

Sample size estimation was based on anticipated effect sizes for systolic blood pressure reduction from previous multidomain interventions [14]. Assuming a clinically meaningful difference of 8 mmHg between groups, with a standard deviation of 10 mmHg, α = 0.05, and power (1 − β) of 80% (β = 0.20), the required sample size was 25 participants per group. Accounting for an anticipated 15% dropout rate, we aimed to recruit 52 participants (26 per group). Post hoc power analysis revealed that our achieved sample sizes provided >99% power to detect the observed effect sizes for primary outcomes.

### 2.3. Randomization and Blinding

Following baseline assessments, the participants were randomized in a 1:1 ratio to the intervention or control group using computer-generated random sequences (Microsoft Excel 2016). Randomization was performed in permuted blocks of variable sizes by an independent researcher who was not involved in participant recruitment, assessment, or intervention delivery. Allocation was concealed using sealed, opaque, sequentially numbered envelopes, which were opened only after the participant had completed all baseline assessments.

Due to the nature of the exercise intervention, participants could not be blinded to group allocation. However, all outcome assessors and data analysts were blinded to participant group assignments throughout the study, and participants were instructed not to disclose their allocation during assessments. This approach was intended to minimize detection and analysis bias.

### 2.4. Intervention Protocol

The intervention group participated in a supervised 18-week multi-dominant outdoor exercise program, consisting of two 60 min sessions per week [26]. Sessions were conducted in groups of 4–6 participants in local parks and outdoor fitness areas. Each session followed a standardized structure based on ACSM guidelines [25]:

**Warm-up (10 min):** Dynamic movements, joint mobility exercises, and activation of major muscle groups.

**Main segment (40 min):** Integrated training comprised the following:

- *Strength training:* Progressive multi-joint exercises with bodyweight and resistance bands, incorporating isometric holds (30–60 s) in all sessions targeting major muscle groups [27].
- *Cardiovascular training:* Interval-based activities including brisk walking, stepping, and functional movements guided by the Borg Rating of Perceived Exertion scale (target intensity: 6–7/10) [24].
- *Balance and coordination:* Static and dynamic balance challenges, proprioceptive training, and functional movement patterns.
- *Cognitive training:* Dual-task exercises combining physical movement with cognitive challenges, memory games, and executive function tasks.

**Cool-down (10 min):** Static stretching, relaxation techniques, and mindfulness exercises incorporating elements of Tai Chi and breathing techniques.

The sessions were supervised by qualified exercise professionals with experience in older adult populations. Progression was individualized based on participant capacity and performance, following established guidelines for exercise prescription in older adults [6].

The participants were also encouraged to engage in independent walking activities to achieve the recommended 150 min of weekly moderate-intensity physical activity [28]. Walking logs were maintained to monitor adherence to home-based activities.

### 2.5. Control Group

The control group participants were instructed to maintain their usual activities and lifestyle patterns without participating in any structured exercise programs during the 18-week intervention period. As part of their involvement, they received general health education materials drawn from the University of Older Adults program. These materials included printed modules on cardiovascular risk factors, balanced nutrition, World Health Organization physical activity recommendations for older adults, fall prevention strategies, and cognitive health maintenance. Content was standardized for all participants and delivered without any supervised or individualized exercise prescription. Upon completion of the study, control group participants were offered the opportunity to take part in the full ACTIVA-Senior intervention program.

### 2.6. Outcome Measures

Detailed descriptions of the outcome measures, including their psychometric properties, standardization procedures, and rationale for selection, are available in the ACTIVA-Senior Protocol Study [24]. The protocol provides comprehensive documentation of the measurement tools, assessment timelines, and quality control procedures used throughout the trial.

#### 2.6.1. Primary Outcomes

Resting blood pressure (systolic and diastolic) was measured using a calibrated Omron M3 automatic monitor following standardized protocols [3]. Three measurements were taken after 5 min of rest in a seated position, with the average of the final two measurements recorded.

Functional capacity was assessed via the 6MWT conducted on a 30 m course following American Thoracic Society guidelines [21].

#### 2.6.2. Secondary Outcomes

Body composition parameters including body fat and visceral fat level were assessed using bioelectrical impedance analysis (Omron HBF-514C).

Resting heart rate was measured concurrently with blood pressure assessment.

All assessments were conducted at baseline and immediately following the 18-week intervention period by trained personnel blinded to group allocation.

#### 2.6.3. Statistical Analysis

Statistical analyses were performed using SPSS version 28.0. Descriptive statistics included means ± standard deviations for continuous variables and frequencies with percentages for categorical variables. Baseline characteristics were compared between groups using independent *t*-tests for continuous variables and chi-square tests for categorical variables.

The primary analysis employed repeated-measures ANOVA to examine Time × Group interactions for all outcomes. Sphericity assumptions were tested using Mauchly’s test, with Greenhouse–Geisser corrections applied when violated. Effect sizes were calculated using partial eta-squared (ηp^2^) with interpretations of small (0.01), medium (0.06), and large (≥0.14) effects. Post hoc comparisons used Bonferroni corrections for multiple testing.

Within-group changes were assessed using paired *t*-tests, with effect sizes calculated using Cohen’s d. Between-group differences in change scores were evaluated using independent *t*-tests. This change score approach was used to account for any baseline variability between groups, ensuring that the analyses focused on relative improvements over time rather than absolute post-intervention values. Clinical significance was determined using established minimal clinically important differences: 5 mmHg for blood pressure and 17.8 m for 6MWT performance in Asian older adult populations [22].

The Number Needed to Treat (NNT) was calculated for clinically meaningful improvements. All analyses were conducted using an intention-to-treat approach where possible, with significance set at *p* < 0.05.

## 3. Results

### 3.1. Participant Flow and Baseline Characteristics

A total of 96 individuals were initially assessed for eligibility, with 52 meeting the inclusion criteria and providing informed consent. Twenty-six participants were randomized to each group. During the 18-week intervention period, six participants withdrew: one from the intervention group (newly diagnosed with cancer) and five from the control group (lack of commitment/motivation). The final analytical sample comprised 46 participants (intervention: n = 25; control: n = 21), representing an overall retention rate of 88.5%.

Baseline characteristics were well balanced between groups (Table 1). The mean age was 66.0 ± 5.1 years, with 58.7% female participants, all of whom were postmenopausal. No significant differences were observed between groups for demographic variables, cardiovascular parameters, or functional capacity measures at baseline (all *p* > 0.05). Notably, the control group demonstrated a higher baseline 6MWT performance (586.4 ± 61.7 vs. 548.9 ± 61.8 m, *p* = 0.046), which was accounted for in subsequent analyses. Although baseline 6MWT performance differed between groups, analysis based on change scores indicated that the intervention group achieved significantly greater improvements than the control group, supporting the robustness of the findings.

### 3.2. Primary Outcomes

The repeated-measures ANOVA revealed significant Time × Group interactions for all primary outcomes (Table 2). For systolic blood pressure, the intervention group demonstrated a mean reduction of 17.4 ± 11.0 mmHg (95% CI: −21.9 to −12.9, *p* < 0.001, Cohen’s d = 1.24), while the control group showed a non-significant reduction of 3.1 ± 7.2 mmHg (95% CI: −6.3 to 0.2, *p* = 0.065). The between-group difference was highly significant (F = 26.49, *p* < 0.001, ηp^2^ = 0.376). Similarly, diastolic blood pressure decreased by 9.2 ± 7.0 mmHg in the intervention group (95% CI: −12.1 to −6.3, *p* < 0.001, Cohen’s d = 1.15) compared to 1.6 ± 6.5 mmHg in the control group (95% CI: −4.5 to 1.4, *p* = 0.281). The Time × Group interaction was significant (F = 14.52, *p* < 0.001, ηp^2^ = 0.248). Functional capacity, as measured by the 6MWT, improved by 64.7 ± 45.6 m in the intervention group (95% CI: 45.9 to 83.5, *p* < 0.001, Cohen’s d = 0.99), while the control group showed a non-significant decline of 16.8 ± 43.6 m (95% CI: −36.6 to 3.1, *p* = 0.093). The between-group difference was highly significant (F = 37.89, *p* < 0.001, ηp^2^ = 0.463)

**Figure 2:**
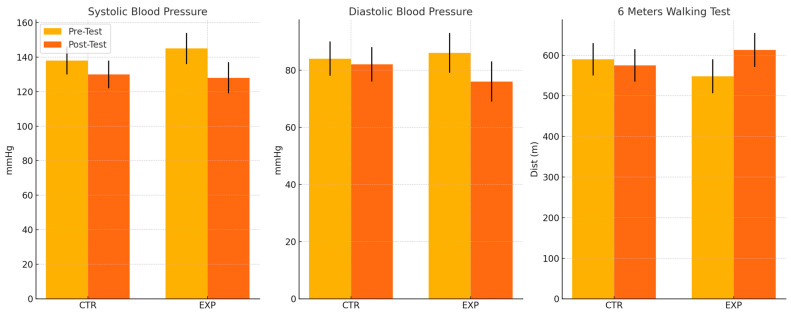
Pre- and post-test comparisons for systolic blood pressure, diastolic blood pressure, and Six-Minute Walk Test by intervention and control groups.

The evolution of key variables over time is illustrated in Figure 2.

### 3.3. Secondary Outcomes

Body composition parameters showed modest but statistically significant improvements in the intervention group. Percent body fat decreased by 1.3 ± 2.2% (95% CI: −2.2 to −0.4, *p* = 0.007, Cohen’s d = 0.19), while visceral fat level decreased by 0.9 ± 1.4 units (95% CI: −1.5 to −0.4, *p* = 0.002, Cohen’s d = 0.24). The control group also demonstrated significant reductions in both parameters, though of smaller magnitude. Between-group differences were not statistically significant for body composition outcomes.

### 3.4. Clinical Significance and Number Needed to Treat

The clinical significance analysis revealed that 84.0% of intervention participants (21/25) achieved clinically meaningful systolic blood pressure reductions of ≥5 mmHg, compared to 38.1% of control participants (8/21). This yielded an absolute risk reduction of 45.9% and an NNT of 2.2, demonstrating exceptional clinical efficiency [3].

For functional capacity, 84.0% of intervention participants (21/25) achieved the minimal clinically important difference for 6MWT improvement (≥17.8 m), compared to 14.3% of control participants (3/21). The absolute risk reduction was 69.7% with an NNT of 1.4.

### 3.5. Adherence and Safety

The main attendance rate for the intervention group was 89.3% (range: 76–100%). No serious adverse events were reported during the study period. Minor events included temporary muscle soreness (n = 3) and minor joint discomfort (n = 2), all of which were resolved within 48 h without intervention, consistent with safety profiles reported in similar outdoor exercise programs [19].

## 4. Discussion

The ACTIVA-Senior study demonstrates that an 18-week multidomain outdoor exercise intervention produces clinically meaningful and statistically significant improvements in cardiovascular health and functional capacity in older adults. The magnitude of blood pressure reductions observed (17.4 mmHg systolic, 9.2 mmHg diastolic) substantially exceeds established thresholds for clinical significance [3] and represents one of the largest effects reported in the multidomain intervention literature.

In relation to cardiovascular outcomes, our findings align with and extend previous research demonstrating the superior efficacy of isometric exercise for blood pressure management [15]. Edwards and colleagues reported that isometric training produced greater systolic blood pressure reductions (−8.6 mmHg) compared to high-intensity interval training (−2.9 mmHg) in adults with elevated blood pressure [29]. However, our observed reduction of 17.4 mmHg considerably exceeds these findings, potentially due to the multidomain nature of our intervention and the inclusion of isometric exercises in every session. Recent meta-analytic evidence from Suematsu and colleagues confirms that isometric resistance training produces superior blood pressure reductions (−11.0 mmHg) compared to other exercise modalities [30].

Our results support and extend these findings, suggesting that integrating isometric exercises within comprehensive multidomain programs may amplify cardiovascular benefits. The mechanisms underlying these effects likely include enhanced endothelium-dependent vasodilation, improved baroreflex sensitivity, and reduced peripheral vascular resistance through chronic adaptation to isometric tension [17]. Aerobic components contribute to vascular compliance and autonomic regulation, while cognitive engagement may reduce sympathetic tone. In addition, the outdoor setting—through its influence on psychological stress, sunlight exposure, and environmental variability—may enhance parasympathetic activity and vascular reactivity [19,20]. These combined effects likely contributed to the large and clinically significant blood pressure reductions observed.

Turning to functional capacity, the 64.7 m improvement in 6MWT performance in the intervention group represents a substantial functional gain, exceeding the minimal clinically important difference of 17.8 m established for Asian older adult populations more than threefold [22].

Multiple physiological pathways likely contributed to this gain. Regular aerobic and resistance training can increase cardiac stroke volume, skeletal muscle oxidative capacity, and neuromuscular efficiency. Balance and coordination components may have enhanced proprioceptive control, reduced gait variability, and increased confidence during ambulation [21]. The cognitive dual-task exercises could also improve executive function related to motor planning and real-world walking performance, especially under multitask conditions. These factors collectively support the large increase observed in 6MWT performance.

With respect to body composition, while body composition changes were modest, the observed reductions in percentage body fat and visceral fat levels are encouraging, particularly given the 18-week intervention duration. Visceral adiposity is strongly associated with cardiovascular risk and metabolic dysfunction in older adults [31]. Even small reductions in visceral fat may contribute to improved cardiometabolic profiles and reduced inflammation. The systematic review by Davis Englund and colleagues demonstrated that fat mass reduction was associated with improved physical performance in older adults, supporting the clinical relevance of our body composition findings [32]. Future studies with longer follow-up periods may reveal more substantial changes in body composition parameters. The absence of a dietary or nutritional intervention likely limited the magnitude of fat loss, as energy balance is a key determinant of body composition change. Additionally, the relatively low exercise frequency (two sessions per week) may not have provided sufficient cumulative energy expenditure to produce substantial changes in fat mass, particularly in participants who maintained pre-existing dietary patterns. Another factor is the use of bioelectrical impedance analysis, which, while practical for field studies, has lower precision and sensitivity compared to dual-energy X-ray absorptiometry (DEXA) in detecting subtle changes in fat distribution. These methodological and design factors may explain why body composition changes, while present, were not statistically superior to those in the control group, despite clear functional and cardiovascular benefits.

These findings are supported by several plausible physiological mechanisms. The superior efficacy of our multidomain approach likely reflects synergistic effects across multiple physiological systems. Isometric exercises enhance vascular function through nitric oxide-mediated vasodilation and improved endothelial function [16]. Aerobic training contributes to enhanced cardiovascular efficiency and autonomic function. Balance and cognitive training may provide additional benefits through neural adaptation and improved motor control. The outdoor setting may amplify these benefits through multiple mechanisms [19]. Exposure to natural environments reduces stress hormones, enhances mood, and may improve adherence compared to facility-based programs. Additionally, outdoor exercise may provide greater variability in environmental stimulus, potentially enhancing adaptability and functional capacity.

The clinical and public health significance of our findings extend beyond statistical considerations. With NNT values of 2.2 for meaningful blood pressure reduction and 1.4 for functional improvement, the ACTIVA-Senior intervention demonstrates exceptional clinical efficiency. These values compare favorably to many pharmacological interventions and suggest that multidomain outdoor programs could serve as first-line interventions for cardiovascular health promotion in older adults [3]. From a public health perspective, outdoor multidomain programs offer scalable, cost-effective solutions for population-level interventions [33]. The minimal equipment requirements, community-based delivery model, and potential for group implementation make this approach highly suitable for widespread adoption. Healthcare systems could potentially realize significant cost savings through reduced cardiovascular events and delayed functional decline.

In terms of practical applications, the ACTIVA-Senior multidomain outdoor exercise program can be implemented in community settings with minimal cost and infrastructure. The program requires only basic portable equipment and can be delivered in public spaces, such as parks or outdoor fitness areas, making it widely accessible to older adults. The twice-weekly, 60 min format aligns with WHO physical activity guidelines for older adults and can be adapted to different fitness levels. Qualified exercise professionals can deliver the intervention following the structured session plan outlined in our published protocol [24]. The high adherence rates and absence of serious adverse events in our study suggest that the program is both safe and acceptable for relatively healthy older adults. From a public health perspective, this scalable model could be integrated into municipal wellness initiatives or national aging strategies to promote cardiovascular health, functional independence, and social engagement. By leveraging outdoor environments, the intervention not only supports physical and cognitive function, but also encourages social interaction and exposure to nature, which may further enhance mental well-being.

Nonetheless, several limitations warrant consideration. First, the relatively small sample size, while adequately powered for primary outcomes, may limit generalizability to diverse populations. Second, the single-blind design introduces potential bias, though the blinding of participants in exercise interventions is methodologically challenging. Third, the 18-week intervention period, while substantial, precludes the assessment of long-term sustainability of benefits. The homogeneous study population (Spanish older adults) may limit generalizability to other ethnic and cultural groups. Moreover, the participants were relatively healthy, independent, and motivated to engage in physical activity, which may have facilitated the high adherence rates and large functional gains observed. Therefore, caution is warranted in extrapolating these findings to frailer, more sedentary, or cognitively impaired populations who may require program modifications and additional support to achieve similar outcomes. Additionally, mechanistic studies investigating the physiological pathways underlying the observed benefits—such as endothelial function, heart rate variability, neurocognitive adaptation, and hormonal changes—would enhance theoretical understanding. Long-term follow-up studies are essential to determine the sustainability of cardiovascular and functional improvements. Cost-effectiveness analyses would further support the public health value of multidomain outdoor interventions [33]. Finally, the investigation of optimal intervention dosing (frequency, duration, intensity) could inform evidence-based program design.

Despite the limitations, our study demonstrates several methodological strengths. The randomized controlled design, comprehensive outcome assessment, high retention rate (88.5%), and rigorous statistical analysis support the validity of our findings. The inclusion of both cardiovascular and functional outcomes provides a comprehensive assessment of intervention effects. Additionally, the calculation of clinically meaningful outcomes (NNT) enhances the practical relevance of our results.

## 5. Conclusions

The ACTIVA-Senior study provides robust evidence that multidomain outdoor exercise interventions result in clinically meaningful improvements in both cardiovascular health and functional capacity in older adults. The observed reductions in blood pressure substantially exceed established clinical thresholds, representing some of the most pronounced effects reported to date in the multidomain intervention literature [30]. Similarly, the significant improvement in 6MWT performance underscores the program’s effectiveness in enhancing functional capacity. These outcome gains, together with high adherence rates and the absence of serious adverse events, support the feasibility and safety of implementing such programs in both community and healthcare settings.

These findings carry important implications for healthy aging strategies and public health policy [34]. Notably, the inclusion of isometric exercise within the multidomain framework appears to offer added cardiovascular benefit, consistent with recent findings from Hejazi et al. [35]. Furthermore, delivering these interventions in outdoor environments may increase accessibility, reduce program costs, and enhance participant engagement. As aging populations continue to challenge healthcare systems globally, scalable, evidence-based solutions such as the ACTIVA-Senior model offer a practical and sustainable approach to promoting cardiovascular health and functional independence in older adults.

## Figures and Tables

**Table 1:** Baseline characteristics of study participants.

Variable	Intervention (n = 25)	Control (n = 21)	*p*-Value
Age (years)	65.6 ± 5.3	66.4 ± 4.9	0.609
Female	16 (64.0%)	11 (52.4%)	0.619
CV medication use	8 (32.0%)	8 (38.1%)	0.777
**Cardiovascular variables**			
Resting HR (bpm)	76.0 ± 9.5	77.9 ± 13.2	0.591
Systolic BP (mmHg)	142.9 ± 15.7	139.2 ± 11.8	0.381
Diastolic BP (mmHg)	85.6 ± 8.6	84.0 ± 6.7	0.495
Resting HR (bpm)	76.0 ± 9.5	77.9 ± 13.2	0.591
6MWT distance (m)	548.9 ± 61.8	586.4 ± 61.7	0.046 *
**Body composition variables**			
Height (cm)	160.4 ± 8.9	161.9 ± 9.2	0.577
Weight (kg)	71.7 ± 11.6	70.1 ± 14.2	0.668
BMI (kg/m^2^)	27.8 ± 3.5	26.5 ± 3.8	0.261
Body fat (%)	36.3 ± 6.8	33.3 ± 7.1	0.153
Visceral fat level	13.1 ± 3.9	11.2 ± 4.3	0.137
**Symptoms**			
Joint discomfort	2 (8%)	1 (5%)	0.600

Values are mean ± SD or n (%). * *p* < 0.05.

**Table 2:** Results of Time × Group interaction analysis for primary outcomes.

Outcome	Group	Pre-Test	Post-Test	Change	95% CI	Effect Size (*d*)	*p*-Value	F	η^2^p
**Systolic BP (mmHg)**	**Intervention**	142.9 ± 15.7	125.5 ± 12.2	−17.4 ± 11.0	[−21.9 to −12.9]	1.24	<0.001 *	26.49	0.376
	**Control**	139.2 ± 11.8	136.2 ± 11.4	−3.1 ± 7.2	[−6.3 to 0.2]	0.26	0.065		
**Diastolic BP (mmHg)**	**Intervention**	85.6 ± 8.6	76.4 ± 7.3	−9.2 ± 7.0	[−12.1 to −6.3]	1.15	<0.001 *	14.52	0.248
	**Control**	84.0 ± 6.7	82.5 ± 6.9	−1.6 ± 6.5	[−4.5 to 1.4]	0.23	0.281		
**6MWT (meters)**	**Intervention**	548.9 ± 61.8	613.6 ± 68.6	64.7 ± 45.6	[45.9 to 83.5]	0.99	<0.001 *	37.89	0.463
	**Control**	586.4 ± 61.7	569.6 ± 67.8	−16.8 ± 43.6	[−36.6 to 3.1]	0.26	0.093		

* *p* < 0.05.

## Data Availability

The data presented in this study are available from the corresponding author upon reasonable request.

## Supplementary Materials

The following supporting information can be downloaded at https://www.mdpi.com/article/10.3390/healthcare13161975/s1: CONSORT 2025 checklist. Reference [36] is cited in the supplementary materials.

## Abbreviations

The following abbreviations are used in this manuscript:

IET	Isometric exercise training
6MWT	Six-Minute Walk Test
ηp^2^	Partial eta square
NNT	Number Needed to Treat
CI	Confidence interval

## References

[1] World Health Organization (2015). World Report on Ageing and Health.

[2] Gaidai O., Cao Y., Loginov S. (2023). Global Cardiovascular Diseases Death Rate Prediction. Curr. Probl. Cardiol..

[3] Whelton P.K., Carey R.M., Aronow W.S., Casey D.E., Collins K.J., Dennison Himmelfarb C., DePalma S.M., Gidding S., Jamerson K.A., Jones D.W. (2018). 2017 ACC/AHA/AAPA/ABC/ACPM/AGS/APhA/ASH/ASPC/NMA/PCNA Guideline for the Prevention, Detection, Evaluation, and Management of High Blood Pressure in Adults: A Report of the American College of Cardiology/American Heart Association Task Force on Clinical Practice Guidelines. Hypertension.

[4] Carrasco-Ribelles L.A., Cabrera-Bean M., Danés-Castells M., Zabaleta-Del-Olmo E., Roso-Llorach A., Violán C. (2023). Contribution of Frailty to Multimorbidity Patterns and Trajectories: Longitudinal Dynamic Cohort Study of Aging People. JMIR Public Health Surveill..

[5] Alizaei Yousefabadi H., Niyazi A., Alaee S., Fathi M., Rahimi G.R.M. (2021). Anti-Inflammatory Effects of Exercise on Metabolic Syndrome Patients: A Systematic Review and Meta-Analysis. Biol. Res. Nurs..

[6] Nelson M.E., Rejeski W.J., Blair S.N., Duncan P.W., Judge J.O., King A.C., Macera C.A., Castaneda-Sceppa C. (2007). Physical Activity and Public Health in Older Adults. Med. Sci. Sports Exerc..

[7] Chen Q., Gao X., Wang C., Zhang P. (2025). Influence of different exercise types on vascular endothelial function in middle-aged and older adults—A systematic review and network meta-analysis. Arch. Gerontol. Geriatr..

[8] Green D.J., Hopman M.T.E., Padilla J., Laughlin M.H., Thijssen D.H.J. (2017). Vascular Adaptation to Exercise in Humans: Role of Hemodynamic Stimuli. Physiol. Rev..

[9] García-Llorente A.M., Casimiro-Andújar A.J., Linhares D.G., Vale R.G.D.S., Marcos-Pardo P.J. (2024). Multidomain interventions for sarcopenia and cognitive flexibility in older adults for promoting healthy aging: A systematic review and meta-analysis of randomized controlled trials. Aging Clin. Exp. Res..

[10] Marcos-Pardo P.J., González-Gálvez N., Vaquero-Cristóbal R., Sagarra-Romero L., López-Vivancos A., Velázquez-Díaz D., García G.M.G., Ponce-González J.G., Esteban-Cornejo I., Jiménez-Pavón D. (2021). Multidomain healthy-age programme. Recomendations for healthy ageing: On behalf of the healthy-age network. Cult. Cienc. Y Deporte.

[11] Montero-Odasso M., van der Velde N., Martin F.C., Petrovic M., Tan M.P., Ryg J., Aguilar-Navarro S., Alexander N.B., Becker C., Blain H. (2022). World guidelines for falls prevention and management for older adults: A global initiative. Age Ageing.

[12] Kim D., Ko Y., Jung A. (2022). Longitudinal effects of exercise according to the World Health Organization guidelines on cognitive function in middle-aged and older adults. Front. Public Health.

[13] Cho S.H., Kang H.J., Park Y.K., Moon S.Y., Hong C.H., Na H.R., Song H.-S., Choi M., Jeong S., Park K.W. (2024). SoUth Korean study to PrEvent cognitive impaiRment and protect BRAIN health through Multidomain interventions via facE-to-facE and video communication plaTforms in mild cognitive impairment (SUPERBRAIN-MEET): Protocol for a Multicenter Randomized Controlled Trial. Dement. Neurocogn. Disord..

[14] Lehtisalo J., Rusanen M., Solomon A., Antikainen R., Laatikainen T., Peltonen M., Strandberg T., Tuomilehto J., Soininen H., Kivipelto M. (2022). Effect of a multi-domain lifestyle intervention on cardiovascular risk in older people: The FINGER trial. Eur. Heart J..

[15] Smart N.A., Way D., Carlson D., Millar P., McGowan C., Swaine I., Baross A., Howden R., Ritti-Dias R., Wiles J. (2019). Effects of isometric resistance training on resting blood pressure. J. Hypertens..

[16] Edwards J.J., Wiles J., O’Driscoll J. (2022). Mechanisms for blood pressure reduction following isometric exercise training: A systematic review and meta-analysis. J. Hypertens..

[17] Taylor K.A., Wiles J.D., Coleman D.A., Leeson P., Sharma R., O’dRiscoll J.M. (2019). Neurohumoral and ambulatory haemodynamic adaptations following isometric exercise training in unmedicated hypertensive patients. J. Hypertens..

[18] Moon S.Y., Hong C.H., Jeong J.H., Park Y.K., Na H.R., Song H.-S., Kim B.C., Park K.W., Park H.K., Choi M. (2021). Facility-based and home-based multidomain interventions including cognitive training, exercise, diet, vascular risk management, and motivation for older adults: A randomized controlled feasibility trial. Aging.

[19] Marcos-Pardo P.J., Espeso-García A., Abelleira-Lamela T., Machado D.R.L. (2023). Optimizing outdoor fitness equipment training for older adults: Benefits and future directions for healthy aging. Exp. Gerontol..

[20] Noseworthy M., Peddie L., Buckler E.J., Park F., Pham M., Pratt S., Singh A., Puterman E., Liu-Ambrose T. (2023). The Effects of Outdoor versus Indoor Exercise on Psychological Health, Physical Health, and Physical Activity Behaviour: A Systematic Review of Longitudinal Trials. Int. J. Environ. Res. Public Health.

[21] Giacomantonio N., Morrison P., Rasmussen R., MacKay-Lyons M.J. (2020). Reliability and Validity of the 6-Minute Step Test for Clinical Assessment of Cardiorespiratory Fitness in People at Risk of Cardiovascular Disease. J. Strength Cond. Res..

[22] Liu L., Ma M., Yang X., Yang Y., Huang X., Meng L., Ming D. (2021). Impact of gender and age on 6-Minute Walking Test performance of patients with coronary heart disease compared to healthy elders. Proceedings of the 2021 43rd Annual International Conference of the IEEE Engineering in Medicine & Biology Society (EMBC).

[23] Wang Z., Qi K., Zhang P. (2025). Effect of physical activity interventions on physical and mental health of the elderly: A systematic review and meta-analysis. Aging Clin. Exp. Res..

[24] García-Llorente A.M., Vaquero-Cristóbal R., Casimiro-Andújar A.J., Abraldes J.A., Marcos-Pardo P.J. (2025). ACTIVA-Senior: Study Design and Protocol for a Preliminary Multidomain Outdoor Intervention Promoting Healthy Aging and Mitigating Psycho-Physiological Decline. Healthcare.

[25] Liguori G. (2020). ACSM’s Guidelines for Exercise Testing and Prescription.

[26] Marcos-Pardo P.J., González-Gálvez N., Gea-García G.M., López-Vivancos A., Espeso-García A., Gomes de Souza Vale R. (2020). Sarcopenia as a Mediator of the Effect of a Gerontogymnastics Program on Cardiorespiratory Fitness of Overweight and Obese Older Women: A Randomized Controlled Trial. Int. J. Environ. Res. Public Health.

[27] Fragala M.S., Cadore E.L., Dorgo S., Izquierdo M., Kraemer W.J., Peterson M.D., Ryan E.D. (2019). Resistance Training for Older Adults: Position Statement from the National Strength and Conditioning Association. J. Strength Cond. Res..

[28] Bull F.C., Al-Ansari S.S., Biddle S., Borodulin K., Buman M.P., Cardon G., Carty C., Chaput J.-P., Chastin S., Chou R. (2020). World Health Organization 2020 guidelines on physical activity and sedentary behaviour. Br. J. Sports Med..

[29] Edwards J., De Caux A., Donaldson J., Wiles J., O’DRiscoll J. (2022). Isometric exercise versus high-intensity interval training for the management of blood pressure: A systematic review and meta-analysis. Br. J. Sports Med..

[30] Suematsu Y., Morita H., Abe M., Uehara Y., Koyoshi R., Fujimi K., Ideishi A., Takata K., Kato Y., Hirata T. (2025). Differences in the effects of exercise on blood pressure depending on the physical condition of the subject and the type of exercise: A systematic review and meta-analysis. Hypertens. Res..

[31] Li B., Li Y., Zhang Y., Liu P., Song Y., Zhou Y., Ma L. (2022). Visceral Fat Obesity Correlates with Frailty in Middle-Aged and Older Adults. Diabetes Metab. Syndr. Obes..

[32] Englund D.A., Kirn D.R., Koochek A., Zhu H., Travison T.G., Reid K.F., von Berens Å., Melin M., Cederholm T., Gustafsson T. (2018). Nutritional Supplementation with Physical Activity Improves Muscle Composition in Mobility-Limited Older Adults, the VIVE2 Study: A Randomized, Double-Blind, Placebo-Controlled Trial. J. Gerontol. Ser. A.

[33] Duijvestijn M., de Wit G.A., van Gils P.F., Wendel-Vos G.C.W. (2023). Impact of physical activity on healthcare costs: A systematic review. BMC Health Serv. Res..

[34] Izquierdo M., de Souto Barreto P., Arai H., Bischoff-Ferrari H.A., Cadore E.L., Cesari M., Chen L.-K., Coen P.M., Courneya K.S., Duque G. (2025). Global consensus on optimal exercise recommendations for enhancing healthy longevity in older adults (ICFSR). J. Nutr. Health Aging.

[35] Hejazi K., Iraj Z.A., Saeidi A., Hackney A.C., Laziri F., Suzuki K., Laher I., Hassane Z. (2025). Differential effects of exercise training protocols on blood pressures and lipid profiles in older adults patients with hypertension: A systematic review and meta-analysis. Arch. Gerontol. Geriatr..

[36] Hopewell S., Chan A.W., Collins G.S., Hróbjartsson A., Moher D., Schulz K.F., Tunn R., Aggarwal R., Berkwits M., Berlin J.A. (2025). CONSORT 2025 Statement: Updated guideline for reporting randomised trials. BMJ.

