# Supplementary material for: Impact of a Multidomain Outdoor Exercise Intervention on Cardiovascular Health and Functional Capacity for Healthy Aging: A Randomized Controlled Trial (ACTIVA-Senior Study)
Source: Healthcare (Basel). 2025 Aug 12;13(16):1975. doi: 10.3390/healthcare13161975 (PMC12385504; doi:10.3390/healthcare13161975)
Supplement: Supplementary file 1 [file healthcare-13-01975-s001.zip › healthcare-3802037-supplementary.pdf]

Section/topic	No	CONSORT 2025 checklist item description	Reported on page no.
**Title and abstract:**			
Title and structured abstract	1a	Identification as a randomised trial	p.1
	1b	Structured summary of the trial design, methods, results, and conclusions	p.1
**Open science:**			
Trial registration	2	Name of trial registry, identifying number (with URL) and date of registration	p.4
Protocol and statistical analysis plan	3	Where the trial protocol and statistical analysis plan can be accessed	p.4
Data sharing	4	Where and how the individual de-identified participant data (including data dictionary), statistical code and any other materials can be accessed	p.4
Funding and conflicts of interest	5a	Sources of funding and other support, and role of funders	p.20
	5b	Financial and other conflicts of interest of the manuscript authors	p.20
**Introduction:**			
Background and rationale	6	Scientific background and rationale	pp.2–4
Objectives	7	Specific objectives related to benefits and harms	p.4
**Methods:**			
Patient and public involvement	8	Details of patient or public involvement in the trial	p.4
Trial design	9	Description of trial design including type, allocation ratio, and framework	p.5
Changes to trial protocol	10	Important changes after commencement with reason	p.5
Trial setting	11	Settings and locations of the trial	p.5
Eligibility criteria	12a	Eligibility criteria for participants	pp.5–6
	12b	Eligibility criteria for sites/individuals delivering interventions	p.6
Intervention and comparator	13	Intervention and comparator with sufficient details to allow replication	pp.6–8
Outcomes	14	Prespecified primary and secondary outcomes, including metrics and time points	pp.8–9
Harms	15	How harms were defined and assessed	p.9
Sample size	16a	How sample size was determined	p.9
	16b	Explanation of any interim analyses and stopping guidelines	N/A
**Randomization:**			
Sequence generation	17a	Who generated the random allocation sequence and method used	p.5
	17b	Type of randomization and details of any restriction	p.5

Allocation concealment mechanism	18	Mechanism to implement allocation and conceal sequence	p.5
Implementation	19	Whether enrolment/assignment personnel had access to sequence	p.5
Blinding	20a	Who was blinded after assignment	p.5
	20b	If blinded, how and similarity of interventions	p.5
Statistical methods	21a	Methods used to compare groups for outcomes	pp.9–10
	21b	Definition of who is included in each analysis	p.10
	21c	How missing data were handled	p.10
	21d	Methods for any additional analyses	p.10
**Results:**			
Participant flow			22a
	22b	Losses and exclusions after randomization with reasons	p.10; Fig.1
Recruitment	23a	Dates for recruitment and follow-up	p.5
	23b	Why the trial ended/stopped	p.5
Intervention and comparator delivery	24a	Intervention/comparator as actually administered	pp.6–8
	24b	Concomitant care for each group	p.8
Baseline data	25	Table of baseline characteristics for each group	p.11; Table 1
Numbers analysed, outcomes and estimation	26	For each outcome: numbers, available data, results, effect sizes, precision	pp.11–14; Tables 2–3
Harms	27	All harms or unintended events in each group	p.15
Ancillary analyses	28	Any other analyses, pre-specified or post hoc	pp.14–15
**Discussion:**			
Interpretation			29
Limitations	30	Trial limitations, bias, imprecision, generalizability, multiplicity	pp.18–19

Citation: Hopewell S, Chan AW, Collins GS, Hróbjartsson A, Moher D, Schulz KF, et al. CONSORT 2025 Statement: updated guideline for reporting randomised trials. BMJ. 2025; 388:e081123. https://dx.doi.org/10.1136/bmj-2024-081123
© 2025 Hopewell et al. This is an Open Access article distributed under the terms of the Creative Commons Attribution License (https://creativecommons.org/licenses/by/4.0/), which permits unrestricted use, distribution, and reproduction in any medium, provided the original work is properly cited.

*We strongly recommend reading this statement in conjunction with the CONSORT 2025 Explanation and Elaboration and/or the CONSORT 2025 Expanded Checklist for important clarifications on all the items. We also recommend reading relevant CONSORT extensions. See www.consort-spirit.org.
